# Phosphoproteomics Reveal New Candidates in Abnormal Spermatogenesis of Pseudomales in *Cynoglossus semilaevis*

**DOI:** 10.3390/ijms241411430

**Published:** 2023-07-13

**Authors:** Xihong Li, Lu Li, Zhongkai Cui, Ming Li, Wenteng Xu

**Affiliations:** 1 Function Laboratory for Marine Science and Food Production Process, Laoshan Laboratory, Yellow Sea Fisheries Research Institute, Chinese Academy of Fishery Sciences (CAFS), Qingdao 266071, China; lixh@ysfri.ac.cn (X.L.); 13710179114@139.com (L.L.); cuizk3@foxmail.com (Z.C.); liming@ysfri.ac.cn (M.L.); 2 School of Fishery, Zhejiang Ocean University, Zhoushan 316022, China

**Keywords:** *Cynoglossus semilaevis*, phosphoproteome, spermatogenesis, RanBP2, Mapk

## Abstract

Phosphorylation is a post-translational modification that contributes to versatile protein functions in spermatogenesis, and the variations they generate usually results in abnormal spermatogenesis or sperm dysfunction. The sex-reversal phenomenon exists in Chinese tongue sole under certain conditions such that individuals with a ZW genotype can acquire a male phenotype and are thus called pseudomales. Pseudomale tongue sole can reach sexual maturity but produce only Z-type sperm, and the Z sperm carries paternal epigenetic information. Whether phosphorylation plays a role in the sperm abnormality of pseudomales is unknown. In this study, a phosphoproteomic analysis was performed to compare protein phosphorylation profiles between pseudomale and male testes. Altogether, we identified 14,253 phosphopeptides matching with 4843 proteins, with 1329 differentially phosphorylated peptides corresponding to 1045 differentially phosphorylated proteins (DPPs). Phosphorylation at 781 sites was upregulated and at 548 sites was downregulated. Four motifs were identified among differentially phosphorylated peptides, which were “SP”, “SD”, “RxxS”, and “TP”. Gene Ontology (GO) and Kyoto Encyclopedia of Genes and Genomes (KEGG) analyses suggested that the cell cycle and DNA/RNA processing were significantly enriched with the genes encoding DPPs. To analyze DPP function in depth, a protein-protein interaction network was constructed, and Ran-binding protein 2 was found to play a central role in spermatogenesis by regulating several processes such as the cell cycle, eukaryotic translation, ubiquitination, and minichromosome maintenance. In kinase-associated network analyses, two “mitogen-activated protein kinase (Mapk)-centered” clusters were identified that may account for abnormal spermatogenesis in pseudomales. One cluster was centered on Mapk6, which predominantly regulated the cell cycle by interacting with several cyclin-dependent kinases, and the other was centered on the “testis-expressed kinase 1-like (Tesk1l)/Pim1l-Mapk4l- testis-expressed 14 (Tex14)” kinase cascade, which might contribute to spermatogenesis by regulating β-catenin. Taken together, these data suggested the new candidates involved in pseudomale sperm abnormalities and provided clues to discover the phosphorylated regulatory mechanism underlying tongue sole spermatogenesis.

## 1. Introduction

Sexual size dimorphism (SSD) refers to the difference in the body size of male and female individuals and is common to many animals [1]. In fish, SSD is reported to be exhibited in more than 600 marine and freshwater species [2,3]. Chinese tongue sole (*Cynoglossus semilaevis*) is an SSD fish species in which females grow much faster and larger than males [4,5]. It is favorable in the aquaculture of China due to its delicious taste, tender flesh, and rich nutrition. However, genetic females can undergo sex reversal and acquire a male phenotype (pseudomale), increasing the male-to-female tongue sole ratio and reducing commercial production [6,7], which becomes a major threat to the sustainability of the species’ farming. Pseudomale tongue sole could be sexually mature but only produce Z-type sperm, and the epigenetic information of Z sperm causes the offspring of pseudomale to be prone to sex reversal. Thus, a deep exploration into the abnormal spermatogenesis in pseudomales will be helpful for the development of the breeding industry.

Epigenetic regulations, including DNA methylation, histone modification, and noncoding RNA (ncRNA), are important for sex development and differentiation [8]. Among epigenetic post-translational modifications (PTMs), phosphorylation is crucial and serves an essential function in male reproduction [9,10]. For example, in *Drosophila*, asymmetric cell division of the male germline is regulated via the phosphorylation of histone H3 at threonine 3 [11]. Additionally, chromatin remodeling during mammalian (e.g., mouse, rat, and human) spermatogenesis is stated to be associated with histone phosphorylation [12,13,14,15]. After septin 12 (SEPT12) phosphorylation knock-in in mice, the organization of the septin-based sperm annulus and sperm movement were both restricted, resulting in decreased male fertility [16,17]. Fish studies have revealed the close involvement of protein phosphorylation during spermatogenesis, spermatozoa maturation, and fertilization [18]. Rainbow trout transferrin has been isolated from seminal plasma and phosphorylated at all detected serine (S), threonine (T), and tyrosine (Y) residues [19]. Teleost small mothers against decapentaplegic (Smad) was activated by phosphorylation and thereby contributed to the regulation of male sexual differentiation and maturation [20,21]. Moreover, different mechanisms of spermatozoa activation can be triggered by different phosphorylation profiles [22,23], indicating a critical regulatory role of protein phosphorylation in various fish species. Investigation of testicular sperm phosphorylation may not only contribute to the clarification of the biological basis underlying gonadal differentiation, but also provide a new perspective to identify biomarkers of sex control regulation in aquaculture fish.

With the rapid development of high-throughput omics techniques, large numbers of molecular components can be systematically and conveniently screened [24,25]. Previous studies of Chinese tongue sole were focused on the identification of sex-related genes or proteins and their epigenetic modulations, such as DNA methylation and ncRNA [6,7,8,26,27], but little is known about the phosphorylation in these species. The application of a phosphoproteomics analysis provides an opportunity for the comprehensive discovery of phosphorylated proteins and the characterization of their associations with translational regulation. In this study, we compared the phosphoproteomes of testes from male and pseudomale *C. semilaevis*, aiming to find the differential phosphoproteins/phosphosites and the enriched signaling pathways. Putative protein-protein interaction (PPI) network was also constructed. The study is expected to lead to the identification of many candidates that can be further examined to identify their functions in spermatogenesis and thus expand our knowledge of the sex development of fish.

## 2. Results

### 2.1. Identification and Quantification of Phosphorylation Sites in Tongue Sole Testis

**Figure 1:**
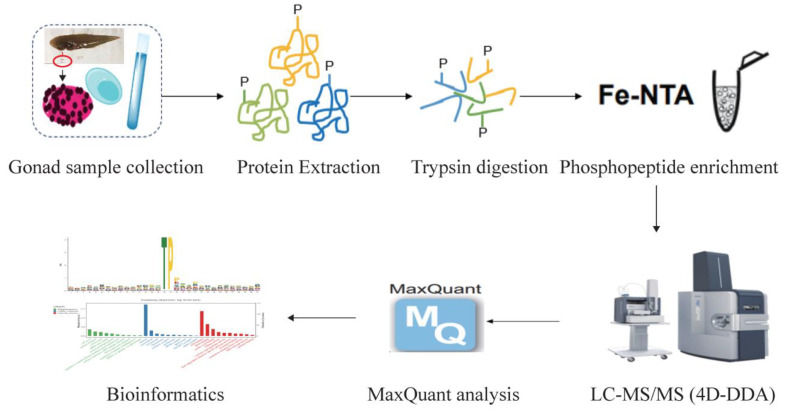
The workflow of the phosphoproteomic analysis.

**Figure 2:**
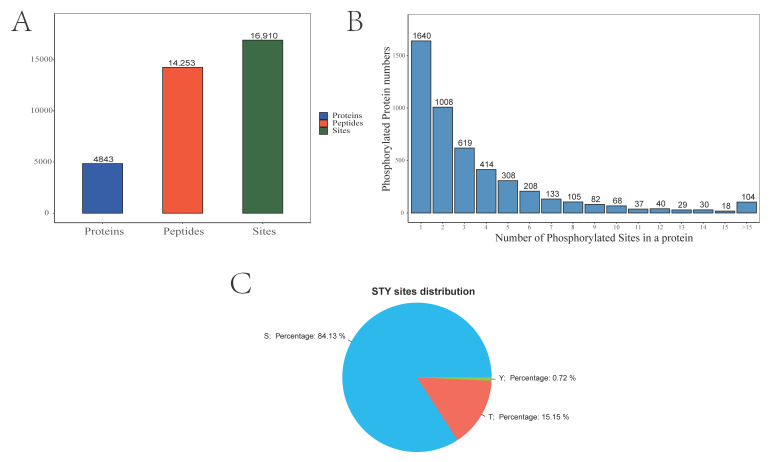
Identification of global phosphorylated proteins and their sites in the testis. (**A**) The number of phosphorylated sites, peptides, and proteins. (**B**) The quantified distribution of phosphorylated sites in proteins. (**C**) The phosphorylation site distribution of serine, threonine, and tyrosine residues.

A four-dimensional (4D) label-free phosphoproteomic analysis including protein extraction, trypsin digestion, phosphopeptide enrichment, liquid chromatography–tandem mass spectrometry (LC-MS/MS) analysis, and data processing (Figure 1) of male and pseudomale testes was accomplished. As shown in Figure 2A and Supplementary Table S1 (Sheets 1–3), a total of 16,910 phosphorylation sites were identified, matching 14,253 peptides and 4843 proteins. The number of phosphorylation sites among the 4843 identified proteins was counted. We found that 1640 proteins carried one phosphorylation site (33.86%), 1008 proteins harbored two phosphorylation sites (20.81%), and 619 proteins carried three phosphorylation sites (12.78%). Moreover, there were 222 proteins that harbored 10–15 phosphorylation sites (4.58%) and 104 proteins that harbored more than 15 phosphorylation sites (2.15%) (Figure 2B). To evaluate the type of phosphorylation sites in tongue sole sperm, the number of serine, threonine, and tyrosine resides among the phosphorylation sites was calculated. 14,227 were located on a serine residue (84.13%), 2561 were located on a threonine residue (15.14%), and 122 were located on a tyrosine residue (0.72%) (Figure 2C).

### 2.2. Differentially Phosphorylated Proteins (DPPs) and Motif Analyses

**Figure 3:**
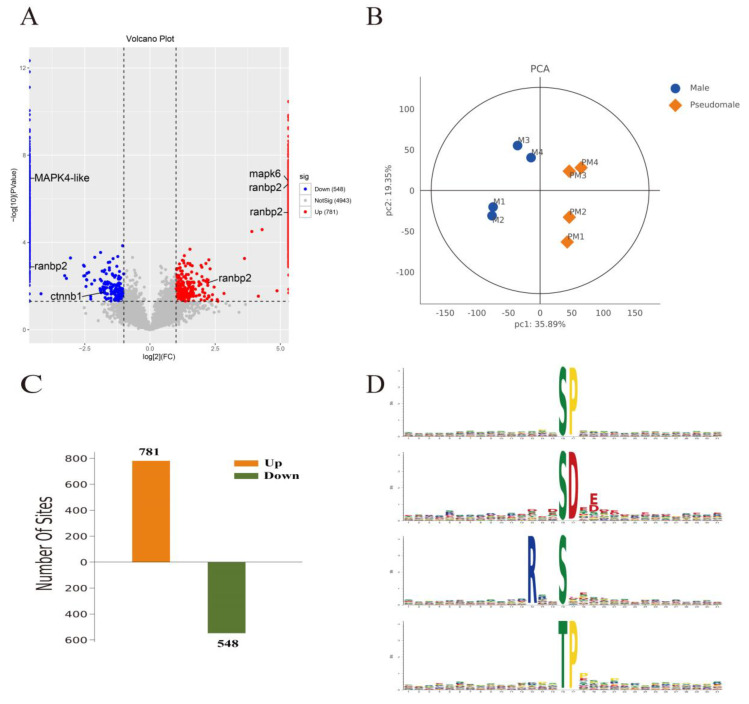
Quantification and motif analysis of differentially phosphorylated proteins (DPPs) between pseudomale and male testis. (**A**) Volcano plot showing the distribution of the relative abundance of phosphopeptides. Blue, red, and gray dots represent hypophoshorylated, hyperphoshorylated, and nondifferentially phosphorylated peptides, respectively. Dotted lines indicate a (±)2-fold change with a *p* < 0.05. The corresponding protein candidates involved in abnormal spermatogenesis are indicated. (**B**) The principal component analysis (PCA) for a total of eight samples of male and pseudomale testes. (**C**) The number of hyper- and hypophosphorylated sites. (**D**) The four signature motifs enriched in the DPPs.

In total, 1329 differentially phosphorylated sites were identified (pseudomale versus male, with a cutoff consisting of a 2-fold change with a *p*-value < 0.05) that corresponded to 1045 proteins. Among these proteins, the phosphorylation of 781 sites was upregulated and the phosphorylation of 548 sites was downregulated (Figure 3; Supplementary Table S1 (Sheet 4)).

To analyze the motif pattern, 31 amino acid sequences were identified with phosphorylation sites located at the central position (position 16). In summary, one threonine-centered and three serine phosphorylation motifs were enriched (Figure 3D). For phosphorylated serine, the most common motifs were “SP”, “SD”, and “RxxS”. For phosphorylated threonine, the motif was “TP”.

### 2.3. Functional Analyses of the DPPs

**Figure 4:**
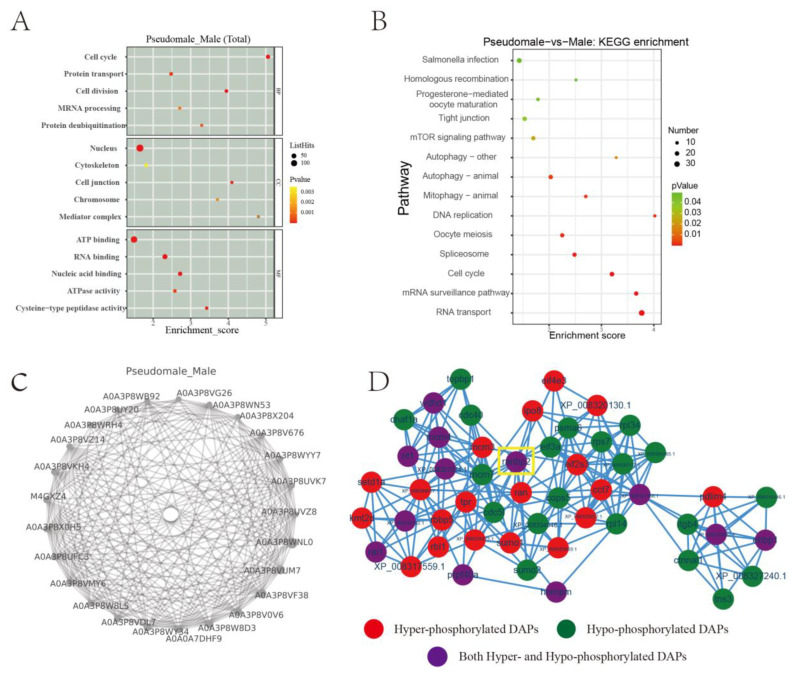
Gene Ontology (GO), Kyoto Encyclopedia of Genes and Genomes (KEGG), and protein-protein interaction analyses of the DPPs: (**A**) GO analysis; (**B**) KEGG enrichment; (**C**) PPI network with the 25 most prevalent proteins. (**D**) The Ran-binding protein (RanBP2) protein interaction network.

As shown in Figure 4A and Supplementary Table S2, Gene Ontology (GO) terms were listed and classified into three categories (biological process, cellular component, and molecular function), and the five terms enriched with DPPs in each category were listed. In the biological process category, the enrichment terms were mainly involved in basic metabolism, such as the cell cycle, protein transport, cell division, and mRNA processing; protein deubiquitination also was included in this category. In the cellular component category, we found that DPPs were predominantly enriched in the nucleus. For molecular function, DPPs were suggested to be closely associated with ATP binding. The Kyoto Encyclopedia of Genes and Genomes (KEGG) enrichment was shown in Figure 4B. Notably, 14 pathways were enriched with DPPs (*p* < 0.05), with several pathways involved in nucleotide processing such as DNA replication, RNA transport, mRNA surveillance, the spliceosome, and the cell cycle, suggesting that phosphorylation might play a divergent role in regulating DNA/RNA processes.

### 2.4. Protein-Protein Interaction (PPI) Network with Phosphorylated Proteins

A PPI network was assembled in order to investigate differences in the biological function of DPPs between pseudomale and male testes. As shown in Figure 4C and Table 1, based on the connection density in the PPI, the top five most enriched DPPs were cell division cycle 5-like protein, a nonspecific serine/threonine protein kinase, cyclin-G-associated kinase-like, tyrosine 3-monooxygenase/tryptophan 5-monooxygenase activation protein epsilon, and E3 SUMO-protein ligase Ran-binding protein (RanBP2). Interestingly, four differentially phosphorylated sites were identified in RanBP2: Ser410, Thr922, Ser2331 hyperphosphorylated, and Ser1128 hypophosphorylated (as determined by comparing pseudomale and male testes). Based on this observation, a PPI network centered on RanBP2 was constructed. Among the interaction partners, several categories were identified, such as cell division cycle protein (Cdc5l and Cdc40), minichromosome maintenance complex component (Mcm3, Mcm4, and Mcm7), small ubiquitin-like modifier (Sumo1, Sumo2), and eukaryotic translation initiation factor (Eif2s3, Eif3a, and Eif4e3).

### 2.5. Kinase-Associated Network

**Figure 5:**
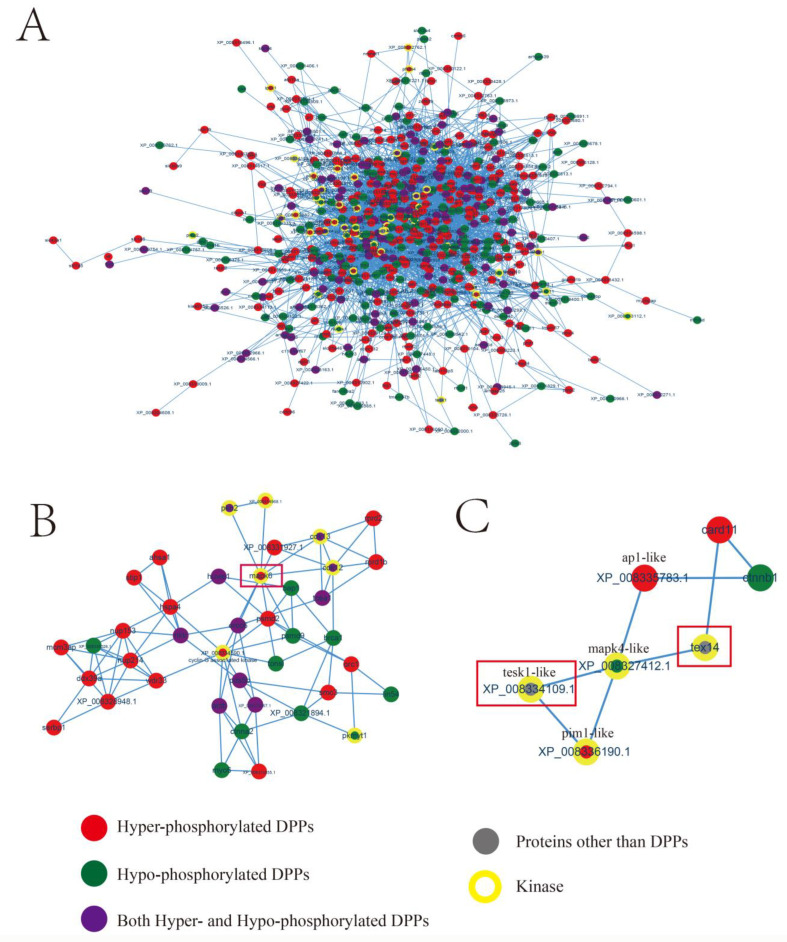
Kinase-associated network. (**A**) Overview of PPI comprising DPPs and four testis-expressed kinases. (**B**) The cluster around Mapk6. (**C**) The cluster containing the Tesk1l/Pim1l-Mapk4l-Tex14 kinase cascade components.

A kinase-associated network was constructed by labeling the kinases in the DPPs and four additional testis-expressed kinases (Tesk1, Tesk1-like, Tesk2, and Tex14), three of which were in the network (Tesk1, Tesk1-like, and Tex14; see Figure 5A and Supplementary Table S2). Eighteen clusters were generated, and two of these clusters were selected for further analysis. As shown in Figure 5B, the center of one cluster was mitogen-activated protein kinase 6 (Mapk6), which interacts with several cyclin-dependent kinases (Cdk12, Cdk13, and cyclin-G-associated kinase-like; XP_008334590.1). The other cluster, “Tesk1l/Pim1l-Mapk4l-Tex14 kinase cascade”, was relatively simple and consisted of seven molecules (Figure 5C), four of which were kinases, including two testis-expressed kinases and three other proteins: caspase recruitment domain-containing protein 11 (Card11), transcription factor Ap1-like (Ap1-like), and β-catenin (Ctnnb).

## 3. Discussion

Sex reversal is very common in lower vertebrates such as fish and reptiles, especially in aquaculture fish. Hormone-induced sex reversal is employed to generate supermale (e.g., YY tilapia) or superfemale (e.g., WW turbot) offspring, which in turn produce monosex fry. Chinese tongue sole has ZZ/ZW sex chromosomes, and the pseudomale should in theory produce both Z and W sperms. However, the absence of W sperm and epigenetic modification related to the Z sperm abrogate the breeding potential of pseudomales, but the mechanism is unknown. A number of diseases such as azoospermia and oligozoospermia have been reported in mammals, while the lack of sperm of specific types is an unusual phenomenon in vertebrates. Discovering the mechanism underlying the absence of specific sperm has particular importance in the fish industry because growth differences due to sexual dimorphism are prevalent in fish, and monosex fry are preferentially favored.

As an important post-translational modification, phosphorylation has been frequently reported in spermatogenesis (especially the classic histone modification), and phosphoproteomic techniques have been recently employed to identify the mechanisms underlying male infertility or reproductive defects [28,29]. However, these studies concentrate on mammals, and few focus on fish. Considering our previous study, we have identified a number of spermatogenesis-related genes in Chinese tongue sole that exhibit no significant difference at the transcriptional and translational levels between males and pseudomales. In this study, we compared the phosphoproteomic patterns between pseudomale and male testes and identified 1045 DPPs that were found to be enriched in the cell cycle and RNA and DNA processing. In fact, the cell cycle is a complex and highly orchestrated process, and its modification leads to severe defects. A recent study suggested that an altered cell cycle was the cause of abnormal meiosis in sterile triploid cyprinid fish [30]. In Chinese tongue sole, abnormal meiosis in pseudomales has also been reported on the basis of a single-cell atlas [31]; therefore, the cell cycle could be a focus of our further analysis.

**Figure 6:**
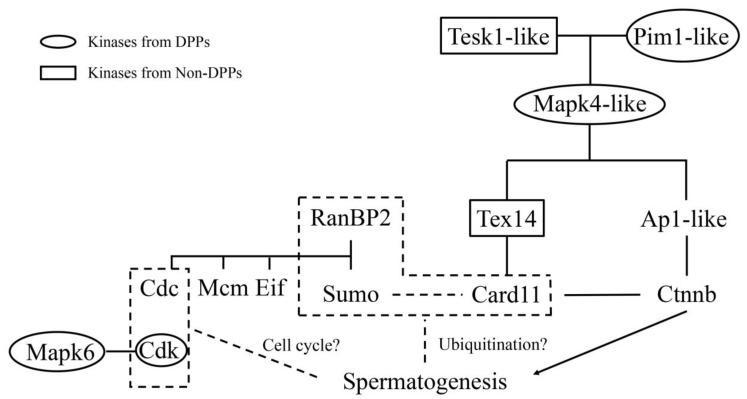
Schematic model of different kinase cascades between pseudomale and male spermatogenesis (experimental proof required).

In a PPI analysis, RanBP2 was identified as a central molecule that participated in the cell cycle by interacting with several cell cycle proteins (Cdc5l and Cdc40) and was also involved in minichromosome maintenance, ubiquitination, and translation. These four phosphorylated sites in RanBP2 might indicate that the action of this protein is regulated by multiple factors. To identify the key kinases involved in the testis, motif signatures in DPPs were analyzed for DPPs. Four motifs were identified: “SP”, “SD”, “RxxS”, and “TP”. According to a previous report, “SP” is recognized by protein kinase B (Pkb), cGMP-dependent protein kinase (Pkg), Iκ-B kinase (Ikk), ataxia telangiectasia mutated kinase (Atm), Cdc2, cyclin-dependent kinase (Cdk), and Mapk; “SD” is recognized by cAMP-dependent protein kinase (Pka), Pkg, casein kinase I (CkI), casein kinase II (CkII), and Atm; “RxxS” is recognized by Pka, Pkb, Pkc, Pkg, CkI, CkII, Ikk, calmodulin-dependent protein kinase II (CaM-II), and Atm; and “TP” is recognized by Pka, Cdc, Cdk, and Mapk [32]. As each motif was related to multiple kinases and identifying the key kinase was difficult, we created a kinase-associated network. Two clusters (one centered around Mapk6 and one clustered around Mapk4l) attracted our attention (Figure 5). The Mapk6 cluster was closely associated with molecules in the cell cycle, such as Cdc and Cdk. The Mapk4l cluster components formed the “Tesk1l/Pim1l-Mapk4l-Tex14” cascade, which was involved in the activation of two testis-expressed kinases (Tesk1l and Tex14) and thereby regulated β-catenin. Both Tesk1l (referred to as Tesk1 in a previous publication) and β-catenin have been reported to participate in spermatogenesis [33,34]. Based on our previous study, many genes localized on the Z chromosome are involved in spermatogenesis; these genes include *doublesex and mab-3 related transcription factor 1* (*dmrt1*), *tesk1l*, *neuralized E3 ubiquitin protein ligase 3* (*neurl3*), and *ring finger and CHY zinc finger domain containing 1* (*rchy1*) [7,35,36]. A Z-linked gene might orchestrate spermatogenesis via epigenetic modification. For example, *dmrt1* and *neurl3* expression is controlled by methylation, which differ between males and pseudomales [7,36]. As ubiquitination ligases, *neurl3* and *rchy1* might participate in spermatogenesis by regulating ubiquitination [35,36]. In addition, *tesk1l* was previously suggested to be involved in spermatogenesis, and it was identified in the “Tesk1l/Pim1l-Mapk4l-Tex14” cascade in this study [33]. Figure 6 shows a schematic model of the hypothetical complex cascade. The Mapk6 cluster predominantly regulates the cell cycle, while the Mapk4 cluster exerts its effect by regulating β-catenin (shown as two bands in an initial Western blot analysis that might correspond to its unphosphorylated and phosphorylated β-catenin). In addition, RanBP2 might mediate ubiquitination and other processes to regulate spermatogenesis. Considering these observations, we will focus on two aspects of phosphorylation in sex reversal in the future. Among kinases, the targets of Mapk4l, Mapk6, and Tesk1l will be screened. On the other hand, the effect of post-translational modification on β-catenin and RanBP2 expression will be clarified. In addition, we have initiated comparative proteomic and other post-translational modification studies between male and pseudomale tongue sole, which might be used to provide a thorough overview in abnormal spermatogenesis.

## 4. Materials and Methods

### 4.1. Experimental Fish and Sex Identification

Chinese tongue sole was obtained from Weizhuo Company (Tangshan, China). To clarify the fish genotype and phenotype, the caudal fin was clipped from each individual, the DNA was isolated, and the sex was determined via a previously reported method [4,37]. Four male and pseudomale individuals were selected for analysis. The average body weight, length, and width of the males were 52.43 g, 20.0 cm, and 5.9 cm, whereas they were 56.03 g, 21.6 cm, and 6.3 cm for pseudomales, respectively.

### 4.2. Protein Preparation and Trypsin Digestion

The phosphoproteomic analyses were carried out by the OE company (Shanghai, China). As shown in Figure 1, gonads from four male and four pseudomale *C. semilaevis* individuals were used for phosphoproteomic analysis. Protein was extracted via a phenol extraction method as previously described [38] with slight alterations. In brief, the sample was resuspended in lysis buffer (240 g/L of sucrose, 5.8 g/L of NaCl, 14.6 g/L of EDTA·2Na, 2 g/L of dithiothreitol, and 0.5 M of Tris-HCl) supplemented with 1 mM of PMSF protease inhibitor (Sangon, Shanghai, China) and PhosStop phosphatase inhibitor (Roche, Basel, Switzerland). After sonication and centrifugation, the upper layer was treated with 0.1 M of ammonium acetate in methanol overnight at −20 °C. After centrifugation and washing, the precipitate was dissolved in SDS lysis buffer (Beyotime, Shanghai, China) at room temperature for 3 h. The isolated protein was quantified with a BCA Protein Assay kit (Thermo, MA, USA), and the proteins were separated via 12% SDS-PAGE gel (Supplementary Figure S1).

Subsequently, the total protein was incubated with 5 mM of dithiothreitol (Sangon, Shanghai, China) at 55 °C for 30 min and treated with 10 mM of iodoacetamide (Sangon, Shanghai, China) in the dark at room temperature for 15 min. Then, 6 volumes of acetone was added to the sample (Wokai, Beijing, China), which was precipitated at −20 °C for 4 h and centrifuged at 8000× *g* at 4 °C for 10 min. The precipitate was then redissolved in 50 mM of ammonium (Sangon, Shanghai, China), and 1 mg/mL of trypsin-TPCK (Hualishi, Beijing, China) was added at a 50:1 (protein:trypsin) mass ratio to digest the proteins (0.5 mg total proteins) at 37 °C overnight. After that, the enzymatic digested peptides were desalted with Sep-Pak C18 cartridges (Waters, MA, USA) and dried in a freeze dryer (Scientz, Ningbo, China).

### 4.3. Enrichment of Phosphopeptides

The enrichment of phosphopeptides was carried out using an IMAC Phosphopeptide Enrichment Kit (Thermo, MA, USA) according to the manufacturer’s instructions. Briefly, the peptide dry powder was suspended in 200 µL of Binding/Wash Buffer. The column was placed in a 2 mL centrifuge tube and centrifuged at 1000× *g* for 30 s to remove the storage buffer. It was then equilibrated by adding 200 µL of Binding/Wash Buffer and centrifuging at the same rotational speed, which was repeated once again. For phosphopeptide enrichment, the above-mentioned 200 µL suspended peptide sample was added to the equilibrated column. We then gently tapped the bottom stopper to mix the sample with the resin. After incubation for 30 min, the column was placed in another 2 mL centrifuge tube and centrifuged at 1000× *g* for 30 s (flow-through discarded). The column was washed with 200 µL of Binding/Wash Buffer three times. It was then added to 200 µL of LC-MS-grade water and centrifuged at 1000× *g* for 30 s. Finally, the column was placed in a new centrifuge tube and eluted with 100 µL of elution buffer twice. The eluate was instantly dried using a high-speed vacuum concentrator.

### 4.4. LC-MS/MS Analysis

For each sample, the enriched tryptic peptides were resuspended with 10 μL of 0.1% (volume ratio) formic acid (FA) in H_2_O, and 2 μL was loaded for LC-MS/MS analysis. The peptides were loaded onto a C18 analytical column (75 μm × 25 cm, 1.6 μm, 120 Å; Ionopticks, Melbourne, Australia) and separated utilizing an EASY-nLC 1200 system (Thermo, MA, USA). Mobile phase A contained 0.1% FA, whereas mobile phase B contained 0.1% FA and 80% acetonitrile (ACN). The gradient elution conditions were as follows: 0~66 min, 3–27% mobile phase B; 66~73 min, 27–46% B; 73~84 min, 46–100% B; and 84~90 min, 100% B. Afterwards, the peptides were analyzed for data-dependent acquisition (DDA) on a timsTOF Pro mass spectrometer (Bruker, Karlsruhe, Germany) following previously reported methods [39] with slight alterations. The capillary voltage was 1.5 kV. The accumulation and ramp time was 100 ms, and the scan range was 100–1700 m/z. The ion mobility varied from 0.75 to 1.4 Vs/cm^2^. The total cycle time was set to 1.17 s in the parallel cumulative serial fragmentation (PASEF) program.

### 4.5. Database Search

The acquired raw data were searched with MaxQuant (version 1.6.17.0) against the UniProt *C. semilaevis* database (Taxon ID, 244447) for label-free relative quantification analysis. The false discovery rate (FDR) threshold was set to be less than 1%. Trypsin/P was selected for proteolytic cleavage with no more than 2 missed sites. Peptide mass tolerances for the first and main searches were 20 ppm and 10 ppm, respectively. The mass tolerance for fragment ions was 0.5 Da. Carbamidomethyl (C) was set as a fixed modification. Oxidation (M), acetyl (protein N-terminus), and phosphorylation (S/T/Y) were the variable modifications. 

Moreover, according to the obtained qualitative tables, the phosphorylation sites with the expression value ≥ 50% in any sample were reserved. The sites with the missing value ≤ 50% were filled with the average values of the same sample and filtered to remove the peptide with a localization probability < 0.75 or a delta score < 8. After median normalization and log2-transformation, high-confidence phosphorylation sites were considered. The dataset was uploaded to iProX under accession number IPX0006597002.

### 4.6. Differential Phosphorylation Site Identification and Motif Analysis

The identified high-confidence sites were used to evaluate the difference between different samples, and the results were visualized. The phosphorylation site difference between the male and pseudomale groups with a fold change > 2 and a *p*-value < 0.05 was considered to be significantly different. The volcano plot was drawn using the R package ggplot2 (version 3.3.6). The principal component analysis (PCA) was carried out using Python matplotlib (version 3.5.2).

The online MoMo program with motif-x (version 5.1.0, http://meme-suite.org/tools/momo, accessed on 8 June 2021) was used to identify potential motifs in common phosphopeptides. The motif width was set to be 31 amino acids (15 at both upstream and downstream of the phosphorylation site). The minimum occurrence was set to be 100, and only a motif with a statistical *p*-value less than 10^−6^ was accepted.

### 4.7. Functional Analysis and PPI Network Construction

Functional enrichment analysis of the DPPs was accomplished via GO annotation and KEGG pathway analysis. The protein-protein interaction (PPI) network was predicted through the Search Tool for the Retrieval of Interacting Genes/Proteins (STRING) database (https://string-db.org, accessed on 12 December 2022) and visualized in Cytoscape v1.0.1 software. Four testis-expressed kinases (Tesk1, XP_008321648.1; Tesk1-like, XP_008334109.1; Tesk2, XP_008333172.1; and Tex14, XP_008331712.1) were included to construct the PPI network.

## Figures and Tables

**Table 1:** Information on the 25 most enriched DPPs used in protein-protein interaction network construction.

Accession	Gene ID	Abbreviation	Protein Name	Chromosome
A0A3P8WNL0	103387747	Cdc5l	Cell division cycle 5-like	12
A0A3P8VUM7	103393521	Smg1	Nonspecific serine/threonine protein kinase	17
A0A3P8VF38	103397928		Cyclin-G-associated kinase-like	Z
A0A3P8V0V6	103377805	Ywhae1	Tyrosine 3-monooxygenase/tryptophan 5-monooxygenase activation protein epsilon	4
A0A3P8W8D3	103392282	RanBP2	E3 SUMO-protein ligase RanBP2	16
A0A0A7DHF9	103393304	ActB2	Actin, beta	17
A0A3P8WY34	103378585	Trip12	Thyroid hormone receptor interactor 12	4
A0A3P8VDL7	103397936	Ttf1	Transcription termination factor 1	Z
A0A3P8W8L5	103388903	Ywhaz	Tyrosine 3-monooxygenase/tryptophan 5-monooxygenase activation protein zeta	13
A0A3P8VMY6	103379263	Shank2	SH3 and multiple ankyrin repeat domains 2a	5
A0A3P8UFC3	103382302	ActA2	Actin, alpha 2	8
A0A3P8X0H5	103380891	Hsp90ab1	Heat shock protein HSP 90-beta	7
M4GXZ4	103393291	Ubc9	SUMO-conjugating enzyme UBC9-like	17
A0A3P8VKH4	103381595	/	Serine/threonine-protein phosphatase alpha-2 isoform-like	8
A0A3P8VZ14	103379050	Mapk6	Mitogen-activated protein kinase 6	5
A0A3P8WRH4	103393241	Usp7	Ubiquitin carboxyl-terminal hydrolase 7	17
A0A3P8UY20	103380065	Chd2	Chromodomain-helicase-DNA-binding protein 2	6
A0A3P8WB92	103391196	Hsp4b	Heat shock protein 4b	15
A0A3P8VG26	103381460	Brca1	BRCA1 DNA repair associated	8
A0A3P8V676	103377844	Ppp5c	Serine/threonine-protein phosphatase 5	4
A0A3P8WN53	103378493	Rabgef1l	RAB guanine nucleotide exchange factor (GEF) 1-like	4
A0A3P8X204	103392668	Setd1a	Histone-lysine N-methyltransferase SETD1A	17
A0A3P8WYY7	103395053	Hsp70	Heat shock cognate 70 kDa protein	19
A0A3P8UVK7	103377749	Usp32	Ubiquitin specific peptidase 32	4
A0A3P8UVZ8	103378868	Pp1cb	Serine/threonine-protein phosphatase PP1-beta catalytic subunit	1

## Data Availability

The data are contained within the article or Supplementary Materials.

## Supplementary Materials

The supporting information can be downloaded at: https://www.mdpi.com/article/10.3390/ijms241411430/s1.

## References

[1] Fairbairn D.J., Blanckenhorn W.U., Székely T. (2007). Sex, Size and Gender Roles: Evolutionary Studies of Sexual Size Dimorphism.

[2] Horne C.R., Hirst A.G., Atkinson D. (2020). Selection for increased male size predicts variation in sexual size dimorphism among fish species. Proc. Biol. Sci..

[3] Mei J., Gui J.F. (2015). Genetic basis and biotechnological manipulation of sexual dimorphism and sex determination in fish. Sci. China Life Sci..

[4] Chen S.L., Li J., Deng S.P., Tian Y.S., Wang Q.Y., Zhuang Z.M., Sha Z.X., Xu J.Y. (2007). Isolation of female-specific AFLP markers and molecular identification of genetic sex in half-smooth tongue sole (*Cynoglossus semilaevis*). Mar. Biotechnol..

[5] Li Y., Hu Y., Yang Y., Cheng J., Cheng X., Chen S. (2022). Genetic parameter estimates for female proportion in tongue sole (*Cynoglossus semilaevis*). Aquaculture.

[6] Chen S., Zhang G., Shao C., Huang Q., Liu G., Zhang P., Song W., An N., Chalopin D., Volff J.N. (2014). Whole-genome sequence of a flatfish provides insights into ZW sex chromosome evolution and adaptation to a benthic lifestyle. Nat. Genet..

[7] Shao C., Li Q., Chen S., Zhang P., Lian J., Hu Q., Sun B., Jin L., Liu S., Wang Z. (2014). Epigenetic modification and inheritance in sexual reversal of fish. Genome Res..

[8] Tang L., Huang F., You W., Poetsch A., Nobrega R.H., Power D.M., Zhu T., Liu K., Wang H.Y., Wang Q. (2022). ceRNA crosstalk mediated by ncRNAs is a novel regulatory mechanism in fish sex determination and differentiation. Genome Res..

[9] Mao B., Zhang W., Zheng Y., Li D., Chen M.Y., Wang Y.F. (2022). Comparative phosphoproteomics reveal new candidates in the regulation of spermatogenesis of *Drosophila melanogaster*. Insect Sci..

[10] Zhang R., Liang C., Guo X., Bao P., Pei J., Wu F., Yin M., Chu M., Yan P. (2022). Quantitative phosphoproteomics analyses reveal the regulatory mechanisms related to frozen-thawed sperm capacitation and acrosome reaction in yak (*Bos grunniens*). Front. Physiol..

[11] Xie J., Wooten M., Tran V., Chen B.C., Pozmanter C., Simbolon C., Betzig E., Chen X. (2015). Histone H3 Threonine Phosphorylation Regulates Asymmetric Histone Inheritance in the Drosophila Male Germline. Cell.

[12] Rogakou E.P., Pilch D.R., Orr A.H., Ivanova V.S., Bonner W.M. (1998). DNA double-stranded breaks induce histone H2AX phosphorylation on serine 139. J. Biol. Chem..

[13] Meyer-Ficca M.L., Scherthan H., Burkle A., Meyer R.G. (2005). Poly(ADP-ribosyl)ation during chromatin remodeling steps in rat spermiogenesis. Chromosoma.

[14] Lu S., Xie Y.M., Li X., Luo J., Shi X.Q., Hong X., Pan Y.H., Ma X. (2009). Mass spectrometry analysis of dynamic post-translational modifications of TH2B during spermatogenesis. Mol. Hum. Reprod..

[15] Zhang Z., Kang X., Mu S. (2014). Histone phosphorylation and spermatogenesis. Yi Chuan = Hered..

[16] Shen Y.R., Wang H.Y., Kuo Y.C., Shih S.C., Hsu C.H., Chen Y.R., Wu S.R., Wang C.Y., Kuo P.L. (2017). SEPT12 phosphorylation results in loss of the septin ring/sperm annulus, defective sperm motility and poor male fertility. PLoS Genet..

[17] Lin C.H., Shen Y.R., Wang H.Y., Chiang C.W., Wang C.Y., Kuo P.L. (2019). Regulation of septin phosphorylation: SEPT12 phosphorylation in sperm septin assembly. Cytoskeleton.

[18] Dietrich M.A., Nynca J., Ciereszko A. (2019). Proteomic and metabolomic insights into the functions of the male reproductive system in fishes. Theriogenology.

[19] Nynca J., Dietrich M.A., Adamek M., Steinhagen D., Bilinska B., Hejmej A., Ciereszko A. (2017). Purification, characterization and expression of transferrin from rainbow trout seminal plasma. Comp. Biochem. Physiol. B Biochem. Mol. Biol..

[20] di Clemente N., Jamin S.P., Lugovskoy A., Carmillo P., Ehrenfels C., Picard J.Y., Whitty A., Josso N., Pepinsky R.B., Cate R.L. (2010). Processing of anti-mullerian hormone regulates receptor activation by a mechanism distinct from TGF-beta. Mol. Endocrinol..

[21] Duan W., Gao F.X., Chen Z.W., Gao Y., Gui J.F., Zhao Z., Shi Y. (2021). A sex-linked SNP mutation in *amhr2* is responsible for male differentiation in obscure puffer (*Takifugu obscurus*). Mol. Biol. Rep..

[22] Gazo I., Dietrich M.A., Pruliere G., Shaliutina-Kolesova A., Shaliutina O., Cosson J., Chenevert J. (2017). Protein phosphorylation in spermatozoa motility of *Acipenser ruthenus* and *Cyprinus carpio*. Reproduction.

[23] Chauvigne F., Ducat C., Ferre A., Hansen T., Carrascal M., Abian J., Finn R.N., Cerda J. (2021). A multiplier peroxiporin signal transduction pathway powers piscine spermatozoa. Proc. Natl. Acad. Sci. USA.

[24] Schneider M.V., Orchard S. (2011). Omics Technologies, Data, and Bioinformatics Principles. Methods Mol. Biol..

[25] Wang X., Sun S., Cao X., Gao J. (2020). Quantitative Phosphoproteomic Analysis Reveals the Regulatory Networks of Elovl6 on Lipid and Glucose Metabolism in Zebrafish. Int. J. Mol. Sci..

[26] Zhu Y., Li Y., Li H., Wang L., Zhang N., Liu Y., Meng L., Xu X., Dong Z., Wei M. (2019). iTRAQ-based analysis of 17β-estradiol induced proteome in Chinese tongue sole *Cynoglossus semilaevis*. J. Oceanol. Limnol..

[27] Xu W., Cui Z., Wang N., Zhang M., Wang J., Xu X., Liu Y., Chen S. (2021). Transcriptomic analysis revealed gene expression profiles during the sex differentiation of Chinese tongue sole (*Cynoglossus semilaevis*). Comp. Biochem. Physiol. Part D Genom. Proteom..

[28] Cohen P. (2002). The origins of protein phosphorylation. Nat. Cell Biol..

[29] Xu Y., Han Q., Ma C., Wang Y., Zhang P., Li C., Cheng X., Xu H. (2021). Comparative Proteomics and Phosphoproteomics Analysis Reveal the Possible Breed Difference in Yorkshire and Duroc Boar Spermatozoa. Front. Cell Dev. Biol..

[30] Zhang C., Li Q., Zhu L., He W., Yang C., Zhang H., Sun Y., Zhou L., Sun Y., Zhu S. (2021). Abnormal meiosis in fertile and sterile triploid cyprinid fish. Sci. China Life Sci..

[31] Wang H.-Y., Liu X., Chen J.-Y., Huang Y., Lu Y., Tan F., Liu Q., Yang M., Li S., Zhang X. (2023). Single-cell-resolution transcriptome map revealed novel genes involved in testicular germ cell progression and somatic cells specification in Chinese tongue sole with sex reversal. Sci. China Life Sci..

[32] Wang Z.-X., Zhou C.-X., Calderón-Mantilla G., Petsalaki E., He J.-J., Song H.-Y., Elsheikha H.M., Zhu X.-Q. (2019). iTRAQ-Based Global Phosphoproteomics Reveals Novel Molecular Differences Between *Toxoplasma gondii* Strains of Different Genotypes. Front. Cell. Infect. Microbiol..

[33] Meng L., Zhu Y., Zhang N., Liu W., Liu Y., Shao C., Wang N., Chen S. (2014). Cloning and characterization of tesk1, a novel spermatogenesis-related gene, in the tongue sole (*Cynoglossus semilaevis*). PLoS ONE.

[34] Zhu Y., Hu Q., Xu W., Li H., Guo H., Meng L., Wei M., Lu S., Shao C., Wang N. (2017). Identification and analysis of the β-catenin1 gene in half-smooth tongue sole (*Cynoglossus semilaevis*). PLoS ONE.

[35] Sun Y., Zhu Y., Cheng P., Zhang M., Wang N., Cui Z., Wei M., Xu W. (2021). A Z-Linked E3 Ubiquitin Ligase *Cs-rchy1* Is Involved in Gametogenesis in Chinese Tongue Sole, *Cynoglossus semilaevis*. Animals.

[36] Xu W., Li H., Dong Z., Cui Z., Zhang N., Meng L., Zhu Y., Liu Y., Li Y., Guo H. (2016). Ubiquitin ligase gene neurl3 plays a role in spermatogenesis of half-smooth tongue sole (*Cynoglossus semilaevis*) by regulating testis protein ubiquitination. Gene.

[37] Chen S.L., Ji X.S., Shao C.W., Li W.L., Yang J.F., Liang Z., Liao X.L., Xu G.B., Xu Y., Song W.T. (2012). Induction of mitogynogenetic diploids and identification of WW super-female using sex-specific SSR markers in half-smooth tongue sole (*Cynoglossus semilaevis*). Mar. Biotechnol..

[38] Zhang X., Tan B., Zhu D., Dufresne D., Jiang T., Chen S. (2021). Proteomics of Homeobox7 Enhanced Salt Tolerance in Mesembryanthemum crystallinum. Int. J. Mol. Sci..

[39] Akkurt Arslan M., Kolman I., Pionneau C., Chardonnet S., Magny R., Baudouin C., Brignole-Baudouin F., Kessal K. (2022). Proteomic Analysis of Tears and Conjunctival Cells Collected with Schirmer Strips Using timsTOF Pro: Preanalytical Considerations. Metabolites.

