# Supplementary material for: Phosphoproteomics Reveal New Candidates in Abnormal Spermatogenesis of Pseudomales in Cynoglossus semilaevis
Source: Int J Mol Sci. 2023 Jul 13;24(14):11430. doi: 10.3390/ijms241411430 (PMC10380018; doi:10.3390/ijms241411430)
Supplement: Supplementary file 1 [file ijms-24-11430-s001.zip › SM-captions.pdf]

Supplementary Figure S1 SDS-PAGE of the total proteins from testes of males and pseudomales. (See Supplementary Materials)

Supplementary Table S1 Information of differentially phosphorylated peptides and proteins. (See Supplementary Materials)

Supplementary Table S2 Information of differentially phosphorylated peptides corresponding to Figure 4 and 5. (See Supplementary Materials)
