# Supplementary figures and images for: Phosphoproteomics Reveal New Candidates in Abnormal Spermatogenesis of Pseudomales in Cynoglossus semilaevis
Source: Int J Mol Sci. 2023 Jul 13;24(14):11430. doi: 10.3390/ijms241411430 (PMC10380018; doi:10.3390/ijms241411430)

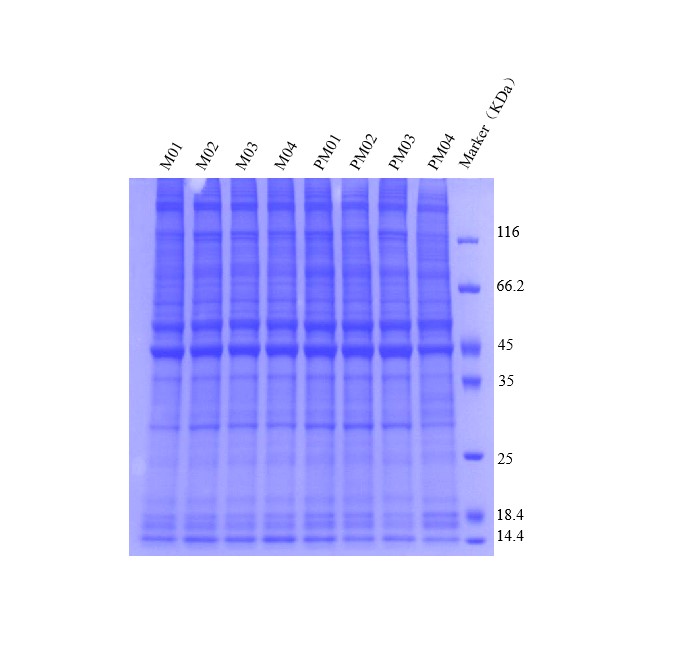

Supplement: Supplementary file 1 [file ijms-24-11430-s001.zip › Supplementary Figure S1.jpg]
